# Procoagulant effects of extracellular vesicles in patients with livedo reticularis

**DOI:** 10.3389/fimmu.2026.1940778

**Published:** 2026-09-18

**Authors:** Avital Baniel, Cristina Ricco, Rohan Dhiman, Ahmed Eldaboush, Caroline Stone, Daniella Furman, Navin Vijayarangan, Darae Kang, Luca Musante, Hossein Fazelinia, Lynn Spruce, Ming-Lin Liu, Victoria P. Werth

**Affiliations:** 1 Department of Dermatology, Veteran Affairs (VA) Medical Center, Philadelphia, PA, United States; 2 Department of Dermatology, University of Pennsylvania (UPENN), Philadelphia, PA, United States; 3 Dermatology Ward, Tel Aviv Sourasky University Medical Center, Tel Aviv, Israel

**Keywords:** coagulation, extracellular vesicles, immunomudulation, livedo reticularis, proteomics, tissue factor

## Abstract

**Background:**

Extracellular vesicles (EVs) are lipid-delimited particles that mediate intercellular communication and have been implicated in both autoimmune and prothrombotic conditions. Livedo reticularis (LR) is a cutaneous vascular pattern associated with impaired dermal arteriolar blood flow and increased vascular risk, yet the contribution of EVs to LR pathogenesis has not been studied.

**Objectives:**

To characterize the procoagulant properties, cellular origin, and proteomic profile of plasma-derived EVs in LR.

**Methods:**

Plasma-derived EVs were isolated from 12 LR patients and seven healthy controls. Tissue factor (TF) activity, EV concentration, proteomic cargo, and cellular origin were assessed using chromogenic assays, nanoparticle tracking analysis, mass spectrometry, and flow cytometry.

**Results:**

TF activity was significantly increased in LR plasma and was enriched within the small EV fraction. Sequential centrifugation demonstrated a stepwise reduction in TF activity, implicating both large and small EVs as contributors. EV concentrations did not differ between groups, indicating qualitative rather than quantitative differences. Proteomic analysis revealed a distinct EV cargo in LR, with widespread protein downregulation and enrichment of coagulation, vascular homeostasis, and VEGF signaling pathways. Notably, KLF8 was markedly reduced. The composition of cells of origin was different, as EVs from LR patients showed an increased leukocyte-derived and decreased platelet-derived profile.

**Conclusion:**

LR is associated with an EV-driven procoagulant and inflammatory phenotype characterized by TF activity, altered cargo, and immune-cell origin, suggesting a role for EV-mediated thrombo-inflammatory microvascular dysfunction in LR pathogenesis. Abundance of KLF8 is markedly reduced, which renders it a target for further mechanistic research.

## Introduction

Extracellular vesicles (EVs) are biologic particles, delimited by a lipid bilayer, that are secreted from cells as a means of autocrine, paracrine, or endocrine messaging (1). It is currently agreed to classify the vesicles by their size. “Small EVs” (sEVs) are largely defined as having a diameter <200 nm, while “large EVs” (lEVs) are >200 nm. Livedo reticularis (LR) is a cutaneous vascular pattern characterized by a transient or persistent, reticular discoloration of the skin resulting from impaired blood flow in the dermal arterioles. Hypercoagulable states are more prevalent in patients with connective tissue disease (CTD) (2), and the presence of LR may signal increased risk for vascular complications in these populations (3). For instance, in systemic lupus erythematosus (SLE), LR is closely tied to antiphospholipid antibody syndrome (APLAS) status. A meta-analysis of 4,810 SLE patients found LR in 25.5% of aPL-positive patients versus 13.3% of aPL-negative patients (4). Earlier data showed an even stronger association: 81% of SLE patients with livedo had elevated anticardiolipin antibodies, and the odds of LR were 23-fold higher in aPL-positive patients, with livedo in this setting linking to Sneddon syndrome (5). Thus, LR is a meaningful marker that identifies a subgroup with aPL positivity and higher thrombotic risk.

In the past, the procoagulant properties of EVs were attributed mainly to the phospholipid membrane of lEVs that are negatively charged and adequately spacious to accommodate the interaction with tissue factor (TF) and the assembly of the components of the coagulation cascade (6, 7). Studies in recent years suggest a role for sEVs as well, showing higher EV concentration, secretion from different cells in patients with (APLAS) (8), and increased TF activity in patients with underlying prothrombotic tendencies such as COVID-19 infection and cancer (9).

While many studies implicate EVs in both the pathogenesis of autoimmunity and CTD as well as hypercoagulative states (10, 11), the role of EVs in LR as a primary cutaneous phenotype has not been investigated. In this study we sought to study the role of plasma-derived EVs in LR.

## Methods

### Patient enrollment

A total of 19 individuals, 12 LR patients and seven HCs, were recruited from the Department of Dermatology at the Hospital of the University of Pennsylvania after signing a written informed consent approved by Penn’s institutional review board. All patients were female, most of whom were Caucasian, with a median age of 40 (IQR: 30.75–52.5). Five had dermatomyositis (DM), three LE, one with overlap of DM and LE, one with overlap of DM and Sjogren’s syndrome, one with rheumatoid arthritis, and one without underlying CTD. At the time blood samples were drawn, 10 patients were treated with immunomodulatory or immunosuppressive medication: eight hydroxychloroquine, four systemic corticosteroids, four mycophenolate mofetil, three methotrexate, two TNF inhibitors, one lenalidomide, one colchicine, and one belimumab. For further details see **Supplementary Table S1**. The controls are not sex- and age-matched.

Patients were included by clinical diagnosis of LR by a board-certified dermatologist, and its presence was ascertained by its typical netlike appearance.

### Plasma preparation and EV isolation

Venous blood was collected with EDTA and centrifuged at 500*g* for 10 min to obtain cell-free plasma. The cell-free plasma was centrifuged at 2,500*g* for 15 min twice to remove the debris and platelets within a few hours after blood collection. Whole blood samples from the healthy volunteers were incubated with LPS for 5h at 37°C to produce high EV-TF activity and were used as positive controls in the TF activity assay. Four ml of the obtained platelet-poor plasma were centrifuged at 20,000*g* for 30 min to remove large EVs (lEV). The collected lEV-depleted supernatants were then loaded on a size-exclusion chromatography column (qEV2, Izon Science) to isolate EVs and centrifuged at 100,000 g for 150 min at 4°C to pellet small EVs (sEVs) and stored at −80°C. Confirmation of the presence and purity of our EV preparations was performed as previously reported (12). Briefly, samples were imaged through a transmission electron microscope. Western blot detected the extramembrane EV marker CD9 and the cytosolic marker TSG101. The negative markers Apo-B100 and calnexin were not detected, and a small amount of ApoA1 was detected.

### Tissue factor activity

Plasma-derived EVs were isolated as mentioned above. TF activity was measured via the Tissue Factor Human Chromogenic Activity Assay Kit (Abcam ab108906, Cambridge, UK).

### Proteomics

Protein content of EVs was analyzed by mass spectrometry using an LC-MS/MS QExactive HF mass spectrometer (Thermo Fisher Scientific, Waltham, MA, USA) coupled with an Ultimate 3000 nano UPLC system and EasySpray source, using data-independent acquisition (DIA). Peptides were identified using Spectronaut software (Biognosys). After preparing the sample and extracting proteins from the EVs, we measured the protein concentration by absorbance at 280 nm. This data allowed us to standardize peptide input across all samples, reducing variability in the mass spectrometer load. Post-data acquisition, we performed median centering normalization on the protein intensities, scaling values within each sample to the median intensity across all samples. This normalization set the median normalized intensity for each sample to zero, preparing data for the pairwise t-test analysis.

### Flow cytometry

EVs were isolated as previously described and characterized by bead-assisted flow cytometry to assess the relative distribution of EVs carrying cell-associated surface markers, in accordance with MISEV2023 recommendations. Briefly, 5 × 10^9^ EVs in 100 µl PBS were incubated with capture beads (Invitrogen, Catalogue #A37304, Carlsbad, CA, USA) under rotation, followed by overnight incubation at 4 °C to enable EV–bead association. Bead-bound EVs were quenched with glycine, blocked with bovine serum albumin, and stained with fluorochrome-conjugated antibodies recognizing EV-associated markers commonly enriched on specific cell types, including CD31 (associated with endothelial cell origin), CD41a (associated with platelet origin), and CD45 (associated with leukocyte origin), together with matched isotype controls. After washing, samples were acquired on a BD FACS Symphony A3 Lite flow cytometer. Machine sets and antibodies used are detailed in **Supplementary Table S2**. Data are reported as the proportion of EVs positive for each marker within the bead-bound EV population and as their relative contribution to the total pool of marker-positive EVs. Hence, these data are interpreted semi-quantitatively as differences in the relative distribution of EV-associated markers between groups rather than as absolute quantification of individual EV populations or definitive determination of cellular origin.

### Statistical analysis

For proteomics, Perseus was used to perform statistical data analysis. For ELISA data Prism 8 (GraphPad Software) was used. For normally distributed data, comparisons between two groups were conducted with the Student t-test, and comparisons among three or more groups were performed by using ANOVA, followed by the Student-Newman-Keuls test. For non-normally distributed data, comparisons between two groups were conducted with the Mann–Whitney test, and comparisons among three or more groups were performed with the Kruskal–Wallis test. Statistical significance was considered at a level of *P*-value < 0.05.

## Results

### Tissue factor activity

TF activity in plasma of LR patients is higher than in HCs (274 ± 53 vs. 140 ± 84 pM). In EVs, increased levels of TF activity were detected in small EVs (84 ± 14 vs. 73 ± 7 pM) but not in large EVs (**Figure 1A**). We analyzed the TF activity of EVs indirectly as well. In the process of sequential centrifugation, we measured TF activity in supernatants after each step. The first supernatant represents plasma without lEVs, and the second represents plasma devoid of both lEVs and sEVs. Supernatants obtained from LR patients exhibited a stepwise reduction in TF activity with successive centrifugation steps, indicating that the removed vesicular fractions contribute to TF activation (**Figure 1B**). These findings support a role for both large and small EVs in mediating TF activity. Notably, this decrease was not seen in supernatants derived from HCs or positive controls. Last, we did not detect a difference in TF activity between large and small EVs (**Supplementary Figure S1**).

**Figure 1:**
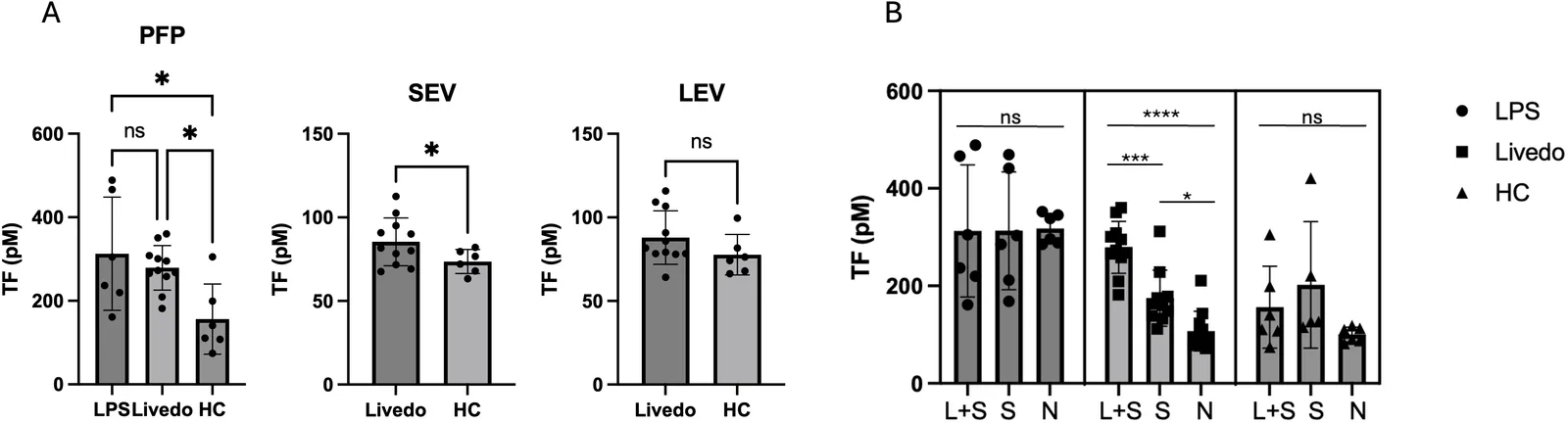
Tissue factor activity. **(A)** In PFP of livedo reticularis patients and positive controls stimulated by LPS, TF activity is higher than in healthy controls **(A)**. In EVs, no significant difference in TF activity was detected, although a clear trend toward higher activity is seen in SEVs. Data bars represent mean. *p < 0.05 between the groups. **(B)** Indirect analysis of TF activity. TF activity was measured in supernatants after each step of centrifugation depletes the plasma from another fraction of EVs. The starting material contains plasma with large and small EVs (L+S), the second step is depleted of large EVs thus contains small EVs only (S), and the third fraction is plasma depleted of both large and small EVs (N). Positive (LPS) and negative (HC) controls show no significant change or pattern in TF activity following sequential depletion of EVs from the plasma, while this pattern is evident in livedo patients. HC, healthy control; LPS, lipopolysaccharide; L+S, large and small EVs; N, No EVs; S, small EVs; TF, tissue factor. Data bars represent median. *p < 0.05, ***p < 0.001, ****p < 0.0001 between the groups.

### EV number

To determine whether the procoagulant properties of EVs in LR patients resulted from increased vesicle production, we quantified EV concentrations using nanoparticle tracking analysis. No significant differences were observed between patients and healthy controls (**Supplementary Figure S2**), implying the procoagulant properties of EVs may not be explained by a higher abundance in our cohort of patients.

### EV protein content

Since the procoagulant properties of EVs may not be explained by a higher abundance, as reported for other procoagulant conditions such as APLAS (13), it may be explained by their cargo. Hence, we sought to analyze the protein content of EVs. We detected 304 differentially expressed proteins (DEPs) between LR and HC samples. Five were upregulated and 269 downregulated in addition to seven proteins detected exclusively in controls (**Figure 2**). LR patients revealed a unique expression pattern compared to HCs, as is demonstrated by principal component analysis (**Figure 3**). LR patients exhibit a clearly distinct and generally downregulated expression pattern compared to HCs. Patients in remission shared the same pattern. A subgroup of patients with profound vasculopathy, including APLAS and Reynaud’s phenomenon, showed expression patterns that were different from LR and controls (**Figure 4**).

**Figure 2:**
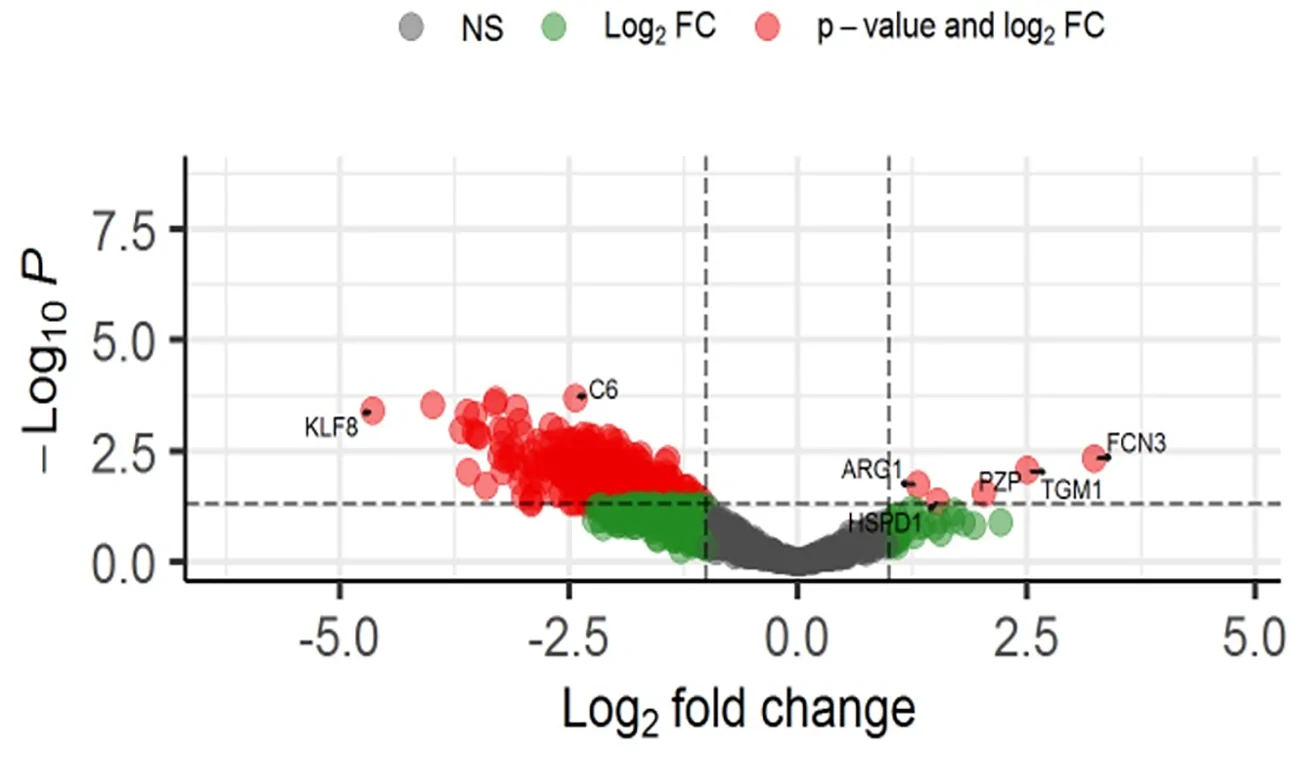
Volcano plot. Proteomic analysis detected 304 differentially expressed proteins between LR and HC samples. Five were upregulated and 269 downregulated. HC, healthy control; LR, livedo reticularis.

**Figure 3:**
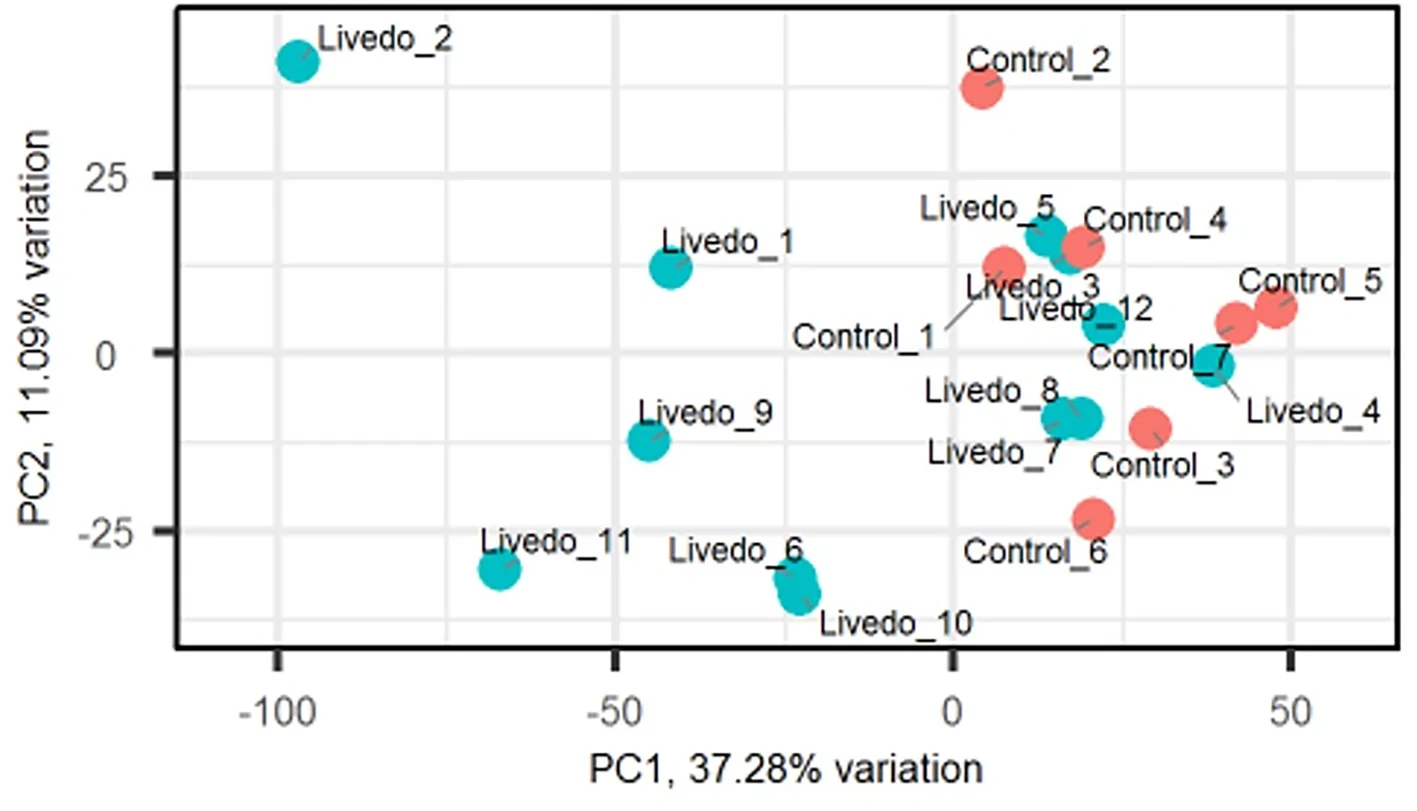
Three-way component analysis demonstrating significant separation in the expression pattern between patients with livedo reticularis and healthy controls.

**Figure 4:**
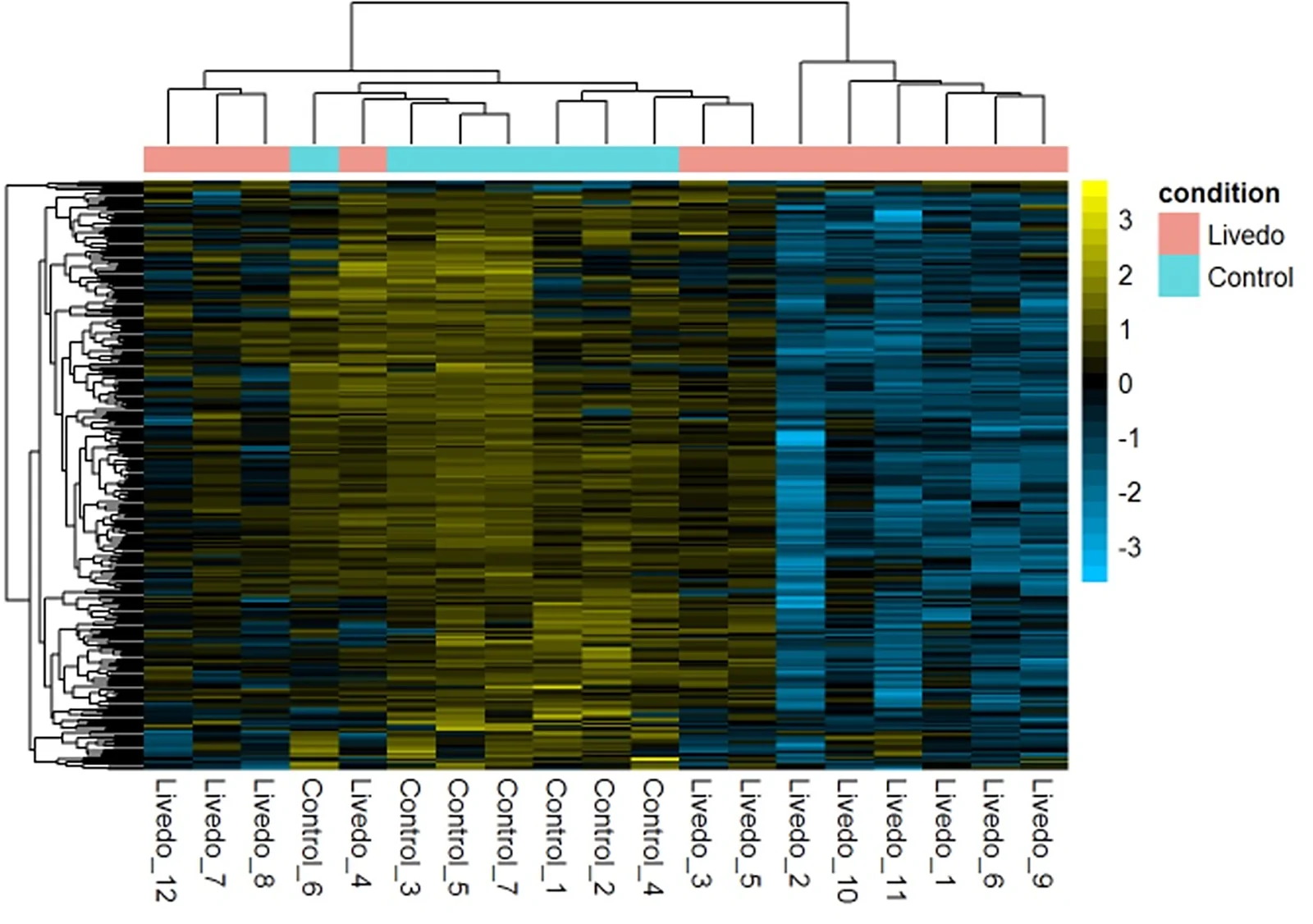
Differentially expression proteins. LR patients display a clearly distinct mostly downregulated expression pattern compared to controls. A subset of patients with profound vasculopathy (Patients 7, 8, 12) display a third pattern and patients in remission (Patients 3, 5) show a pattern similar to controls.

Overrepresentation analysis revealed significant enrichment of proteins associated with homeostasis, coagulation, and vascular endothelial growth factor signaling pathways (**Figure 5**). Amongst the DEPs, Krueppel-like factor 8 (KLF8) exhibited significant prominence, demonstrating a substantial decrease in abundance by a fold change of 4.6 (*p* < 0.001). KLF8, a transcription factor mainly known for its regulatory role in cell cycle and epithelial-mesenchymal transition, is expressed in vascular endothelium and regulates angiogenesis and vessel contractility (14, 15).

**Figure 5:**
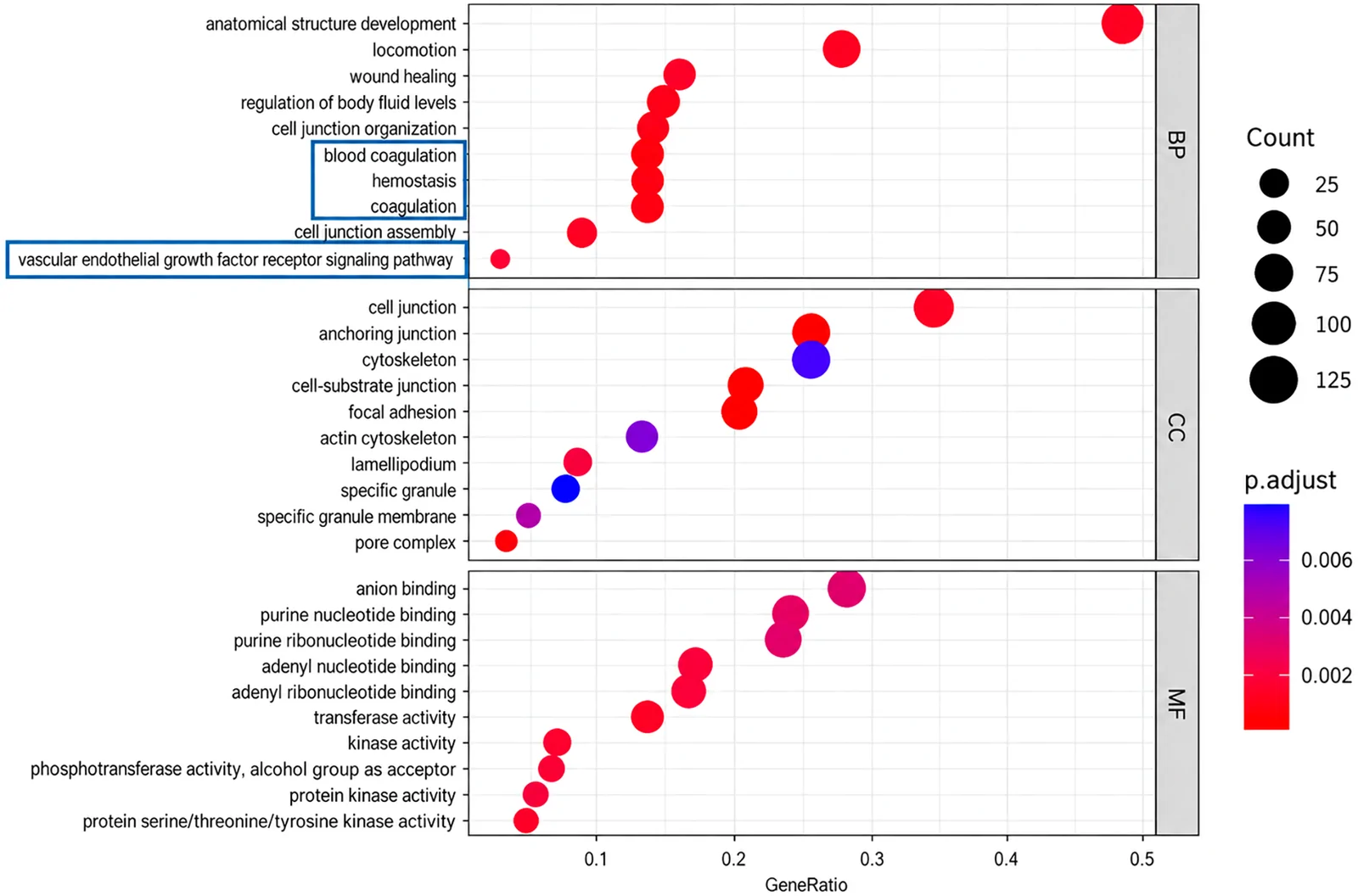
Pathway analysis. Over-representation analysis of the differentially expressed proteins is enriched for pathways related to coagulation, hemostasis, and vascular endothelial growth factor receptor signaling.

### Cellular origin

We investigated the differential production of EVs by cells previously identified as sources of procoagulant EVs. Platelet-derived EVs were the predominant subtype in both LR and control groups, consistent with the established role of platelets as the principal source of circulating EVs. However, our analysis revealed that EVs from patients with LR exhibited an increased presence of leukocyte-derived EVs and a decreased presence of platelet-derived EVs when compared to EVs derived from HCs. No significant changes were observed in endothelial cells (**Figure 6**). This may be indicative of a shift from baseline homeostasis in healthy individuals to an inflammatory EV profile in LR.

**Figure 6:**
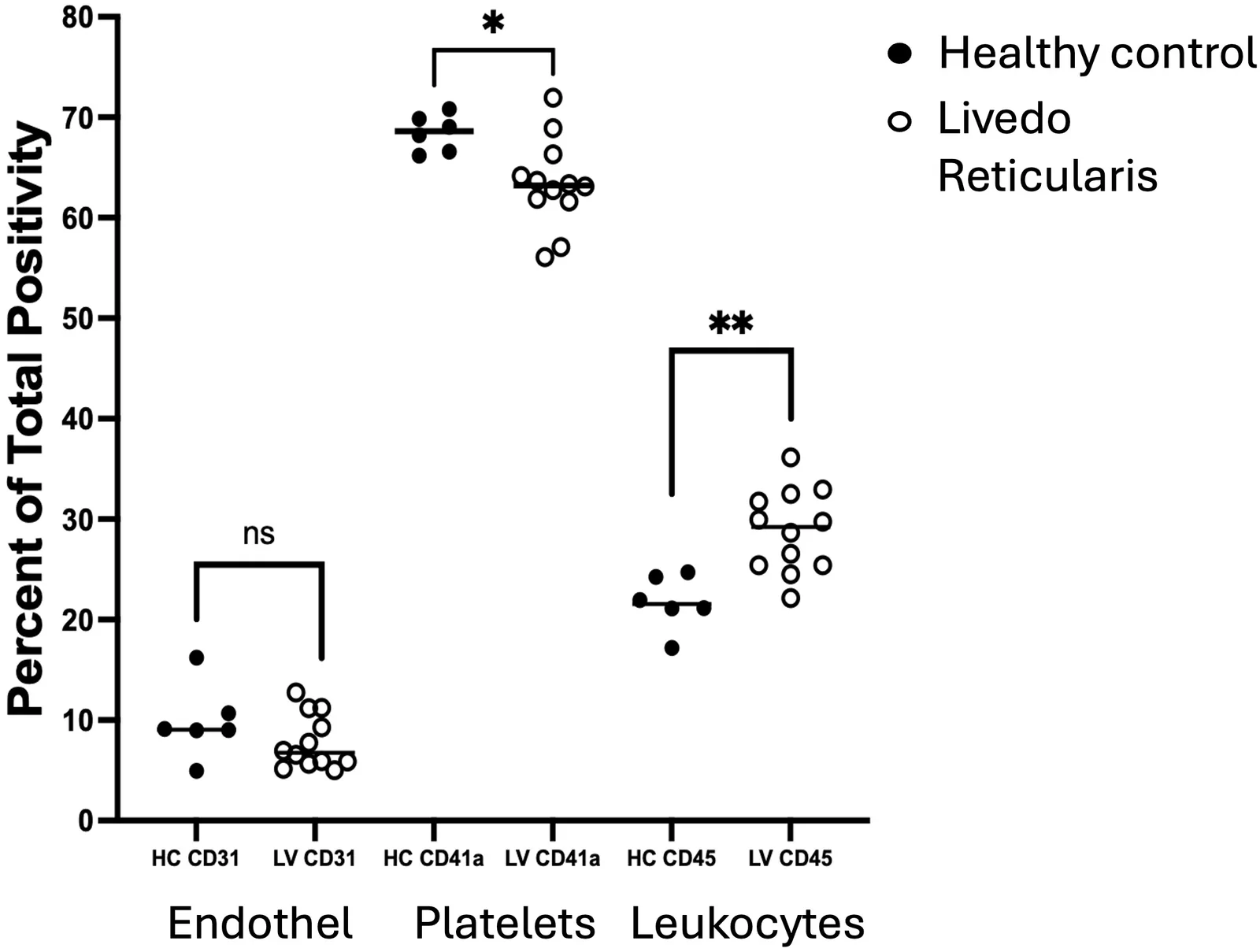
Cellular origin of EVs. LR patients exhibited an increase in leukocyte-derived EVs and a decrease in platelet-derived EVs. There were no significant changes in the fraction of EVs that originated from endothelial cells. EV, extracellular vesicles; LR, livedo reticularis. *p < 0.05, **p < 0.01 between the groups.

## Discussion

Our findings identify a distinct EV–driven procoagulant phenotype in LR that reflects alterations in EV function, cargo, and cellular origin.

TF activity was increased in sEV fractions per direct measurement, and sequential centrifugation of the supernatants indirectly supports the contribution of large and small EVs to TF activity. While TF-bearing EVs are well established as mediators of thrombosis and vascular dysfunction in a variety of diseases, and LR is associated with hypercoagulable states and endothelial dysfunction, to date no study has directly investigated or demonstrated a mechanistic or clinical association between TF activity in EVs and LR as a distinct clinical entity. Our data herein implicates both small and large EVs in the pathogenesis of LR, at least partially in a TF-dependent manner.

Despite comparable EV concentrations between groups, proteomic profiling revealed a profoundly altered EV cargo in LR, characterized by widespread protein downregulation and enrichment of pathways governing coagulation, vascular homeostasis, and VEGF signaling. Although preliminary, these findings suggest that EV cargo alterations may be associated with pathways relevant to the pathogenesis of LR and warrant further mechanistic investigation. Among the differentially expressed proteins, most notable is the reduction in KLF8. Krüppel-like factors are a family of zinc finger transcription factors, currently comprising 18 members, that are characterized by a highly conserved C-terminal domain containing three C2H2-type zinc fingers (16). KLF8 is mainly known for its regulatory role in the cell cycle and epithelial-mesenchymal transition, and when aberrantly expressed, it was found to participate in oncogenic transformation in several tumor types (17–22). In hepatocellular and gastric carcinoma, it induces angiogenesis by binding to the vascular endothelial growth factor promoter (18). In rats, KLF8 was found to affect vessel contractility by regulation of vascular smooth muscle cell (15). To date, KLF8 was not associated with coagulation or thrombosis. Yet, interestingly, downregulation of another member of the KLF family of transcription factors (KLF11) was found to promote thrombosis via loss of inhibition of TF expression (23). Given the substantial functional redundancy among KLF genes throughout evolution (21), the loss of KLF8’s inhibitory effect on TF expression might mechanistically contribute to the vascular dysregulation underlying LR. Another noteworthy observation emerges from a genome-wide transcriptomic analysis of healthy human tissues, in which the skin exhibits the highest expression levels of *KLF8* among all examined organs (24). While intriguing, the potential role of KLF8 in the pathogenesis of LR remains speculative at this stage, and studies to validate its reduced abundance in an independent patient cohort are ongoing. Finally, EV cellular origin analysis demonstrated a shift toward leukocyte-derived EVs with reduced platelet-derived EVs, suggesting a transition from physiological homeostatic balance to an inflammatory EV profile. Interestingly, the cell of origin had been previously shown to affect EVs procoagulant activity and promote TF production by monocyte-derived EVs in pathological conditions (25).

A limitation of the study is the fact that the controls are not sex- and age-matched, as age is known to impact the number and function of EVs.

In summary, this study provides initial evidence of a distinct EV-associated procoagulant phenotype in patients with LR, characterized by altered TF activity, substantial changes in EV protein cargo, and a shift toward a more leukocyte-associated EV profile. These findings extend the potential involvement of EVs in vascular disease to LR and suggest that qualitative changes in EV composition and function, rather than increased EV abundance, may be relevant to its vascular phenotype. The observed enrichment of coagulation- and vascular homeostasis-related pathways, together with the marked reduction in KLF8 abundance, identifies potential mechanisms that warrant further investigation. However, whether these alterations contribute directly to the pathogenesis of LR or reflect underlying vascular or inflammatory processes remains to be established. Validation in larger, independent cohorts and mechanistic studies will be required to define the functional significance of these preliminary observations. As the inhibition of KLF8 using small interfering RNA is currently being explored as a therapeutic approach in oncology, further investigation into its role in cutaneous vasculopathic conditions is highly warranted.

## Data Availability

The datasets presented in this study can be found in online repositories. The names of the repository/repositories and accession number(s) can be found below: https://www.ebi.ac.uk/pride/archive/, PXD077162.

## Supplementary material

Supplementary Figure 1 Small and large EVs isolated from patients with livedo reticularis or healthy controls show no difference in TF activity. Difference is seen only in EVs isolated from LPS-induced plasma. HC, healthy controls; L, large EVs; S, small EVs. Data bars represent mean. **p* < 0.05 between the groups.

Supplementary Figure 2 EV count. EV concentrations were quantified using nanoparticle tracking analysis. EV concentration was not different between the patients and controls in either small or large EVs. EV, extracellular vesicles. Data bars represent mean.

## References

[1] Welsh JA Goberdhan DCI O'Driscoll L Buzas EI Blenkiron C Bussolati B . Minimal information for studies of extracellular vesicles (MISEV2023): from basic to advanced approaches. J Extracell Vesicles. (2024) 13:e12404. doi: 10.1002/jev2.12404 38326288 PMC10850029

[2] Marzo C Solanich T Tassies D Pina E Antonio Rebollo M Gallardo E . Consensus on the management of the clinical challenges of venous thromboembolism in special situations. Thromb Haemost. (2025) 125:944–59. doi: 10.1055/a-2595-1811 40359988 PMC12457007

[3] Toubi E Krause I Fraser A Lev S Stojanovich L Rovensky J . Livedo reticularis is a marker for predicting multi-system thrombosis in antiphospholipid syndrome. Clin Exp Rheumatol. (2005) 23:499–504. 16095119

[4] Loiseau P Foret T DeFilippis EM Risse J Etienne AD Dufrost V . Risk of livedo with antiphospholipid antibodies in patients with systemic lupus erythematosus: A systematic review and meta-analysis. Lupus. (2022) 31:1595–605. doi: 10.1177/09612033221126852 36112747

[5] Englert HJ Loizou S Derue GG Walport MJ Hughes GR . Clinical and immunologic features of livedo reticularis in lupus: a case-control study. Am J Med. (1989) 87:408–10. doi: 10.1016/s0002-9343(89)80823-x 2801732

[6] Chung SM Bae ON Lim KM Noh JY Lee MY Jung YS . Lysophosphatidic acid induces thrombogenic activity through phosphatidylserine exposure and procoagulant microvesicle generation in human erythrocytes. Arterioscler Thromb Vasc Biol. (2007) 27:414–21. doi: 10.1161/01.atv.0000252898.48084.6a 17110600

[7] Mackman N . Microparticles in hemostasis and thrombosis. Circ Res. (2011) 108:1284–97. doi: 10.1161/circresaha.110.233056 21566224 PMC3144708

[8] Štok U Blokar E Lenassi M Holcar M Frank-Bertoncelj M Erman A . Characterization of plasma-derived small extracellular vesicles indicates ongoing endothelial and platelet activation in patients with thrombotic antiphospholipid syndrome. Cells. (2020) 9:1211. doi: 10.3390/cells9051211 PMC7290474 32414170

[9] Sachetto ATA Archibald SJ Hisada Y Rosell A Havervall S van Es N . Tissue factor activity of small and large extracellular vesicles in different diseases. Res Pract Thromb Haemost. (2023) 7:100124. doi: 10.1016/j.rpth.2023.100124 37012986 PMC10015082

[10] Zhao Y Lyu X Wu X Liu Y Zhang N Wei W . Novel perspectives on extracellular vesicles in autoimmune diseases: immunogenicity, inflammation, and immune surveillance. J Clin Invest. (2026) 136:e194715. doi: 10.1172/jci194715 41623176 PMC12867162

[11] Xia C Xu C Liu Y Feng X Le Y Xu H . Extracellular vesicles as orchestrators of arterial and venous thrombosis: a unified mechanistic model. Vascul Pharmacol. (2026) 163:107605. doi: 10.1016/j.vph.2026.107605 41935811

[12] Baniel A Momohara-Ogawa M Bashir M Pandya R Kleitsch J Chin F . Dysregulated pathways and potential biomarkers in dermatomyositis plasma-derived extracellular vesicles: a pilot study. J Invest Dermatol. (2026) 146:541–544.e5. doi: 10.1016/j.jid.2025.06.1597 40716701

[13] Chaturvedi S Alluri R McCrae KR . Extracellular vesicles in the antiphospholipid syndrome. Semin Thromb Hemost. (2018) 44:493–504. doi: 10.1055/s-0037-1599081 28476065

[14] Wang X Zhao J . KLF8 transcription factor participates in oncogenic transformation. Oncogene. (2007) 26:456–61. doi: 10.1038/sj.onc.1209796 16832343

[15] Ha JM Yun SJ Jin SY Lee HS Kim SJ Shin HK . Regulation of vascular smooth muscle phenotype by cross-regulation of Krüppel-like factors. Korean J Physiol Pharmacol. (2017) 21:37–44. doi: 10.4196/kjpp.2017.21.1.37 28066139 PMC5214909

[16] Yuce K Ozkan AI . The Krüppel-like factor (KLF) family, diseases, and physiological events. Gene. (2024) 895:148027. doi: 10.1016/j.gene.2023.148027 38000704

[17] Kumar S Behera A Saha P Srivastava AK . The role of Krüppel-like factor 8 in cancer biology: current research and its clinical relevance. Biochem Pharmacol. (2021) 183:114351. doi: 10.1016/j.bcp.2020.114351 33253644

[18] Cheng S Zhang X Xu Y Dai X Li J Zhang T . Krüppel-like factor 8 regulates VEGFA expression and angiogenesis in hepatocellular carcinoma. Sci Rep. (2018) 8. doi: 10.1038/s41598-018-35786-6 30479372 PMC6258679

[19] Cherukunnath A Davargaon RS Ashraf R Kamdar U Srivastava AK Tripathi PP . KLF8 is activated by TGF-β1 via Smad2 and contributes to ovarian cancer progression. J Cell Biochem. (2022) 123(5):921–34. doi: 10.1002/jcb.30235 35293014

[20] Wang X Zheng M Liu G Xia W McKeown-Longo PJ Hung MC . Krüppel-like factor 8 induces epithelial to mesenchymal transition and epithelial cell invasion. Cancer Res. (2007) 67:7184–93. doi: 10.1158/0008-5472.can-06-4729 17671186

[21] Ilsley MD Gillinder KR Magor GW Huang S Bailey TL Crossley M . Krüppel-like factors compete for promoters and enhancers to fine-tune transcription. Nucleic Acids Res. (2017) 45:6572–88. doi: 10.1093/nar/gkx441 28541545 PMC5499887

[22] Wang WF Li J Du LT Wang LL Yang YM Liu YM . Krüppel-like factor 8 overexpression is correlated with angiogenesis and poor prognosis in gastric cancer. World J Gastroenterol. (2013) 19:4309–15. doi: 10.3748/wjg.v19.i27.4309 23885141 PMC3718898

[23] Liang W Lu H Sun J Zhao G Wang H Guo Y . KLF11 protects against venous thrombosis via suppressing tissue factor expression. Thromb Haemost. (2022) 122:777–88. doi: 10.1055/s-0041-1735191 34428834 PMC10468287

[24] Ilsley MD Gillinder KR Magor GW Huang S Bailey TL Crossley M . Analysis of the human tissue-specific expression by genome-wide integration of transcriptomics and antibody-based proteomics. Mol Cell Proteomics. (2014) 13:397–406. doi: 10.1074/mcp.m113.035600 24309898 PMC3916642

[25] Fagerberg L Hallström BM Oksvold P Kampf C Djureinovic D Odeberg J . Different potential of extracellular vesicles to support thrombin generation: contributions of phosphatidylserine, tissue factor, and cellular origin. Sci Rep. (2017) 7:6522. doi: 10.1038/s41598-017-03262-2 28747771 PMC5529579

