# Supplementary material for: Procoagulant effects of extracellular vesicles in patients with livedo reticularis
Source: Front Immunol. 2026 Sep 18;17:1940778. doi: 10.3389/fimmu.2026.1940778 (PMC13630680; doi:10.3389/fimmu.2026.1940778)
Supplement: Supplementary file 1 [file SupplementaryFile1.pdf]

frontiers

The International Society for the Advancement of Cytometry (ISAC) has highlighted the importance of including comprehensive methodological information to ensure data reproducibility and reliability. In line with this, Frontiers in Immunology now requires authors to submit a checklist for manuscripts that involve flow or mass cytometry. This checklist helps standardize the reporting process, improving the quality and transparency of published data. By doing so, we support scientific progress, making it easier for other researchers to replicate and validate experiments.

This form should be submitted with any manuscripts using flow or mass cytometry.

Sample/specimen/material description

☐ Total blood

☐ PBMCs

☐ Organ digests

Other Plasma-derived extracellular vesicles

Did the samples suffer any treatment before or after incubation with the antibodies?

☐ Drug ___________________________________

☐ Cell permeabilization _____________________

☐ Dye _____________________________________

☐ Propidium iodine

☑ Not applicable

Other _____________________________________

Instrument and antibodies

Name of the Cytometer BD FACS Symphony A3 Lite

**Antibodies and targets**	**Fluorochrome/ Metal**	**Catalog number/Company**
e.g. anti-CD4	FITC	Cat. XXX/ XXX ltd.

Anti-Human CD45	BUV563	BD/#748720/HI30
IgG1 κ Isotype Control	BUV563	BD/#612920/X40
Anti-Human CD41a	BV786	BD/#740975/HIP8
IgG1 k Isotype Control	BV786	BD/#563330/X40
Anti-Human CD31	PE	BD/#555446/WM59
IgG1, κ Isotype Control	PE	BD/#555749/MOPC-21

Data analyses

1. Name of the software FlowJo v10
2. Reference gating strategy in the manuscript or supplementary material

Gating strategy in (eg Figure X) Supplementary figure 3
