# Supplementary material for: Procoagulant effects of extracellular vesicles in patients with livedo reticularis
Source: Front Immunol. 2026 Sep 18;17:1940778. doi: 10.3389/fimmu.2026.1940778 (PMC13630680; doi:10.3389/fimmu.2026.1940778)
Supplement: Supplementary file 3 [file Table2.docx]

| Flow Cytometer Setup | | | | |
| --- | --- | --- | --- | --- |
| Instrument: BD FACS Symphony A3 Lite | | | |  |
| Laser lines | 355nm | | 405nm | 561nm |
| Emission filters | 379/28 | 586/15 | 780/60 | 586/15 |
| Fluorochrome | BUV395 | BUV563 | BV786 | PE |

| Reagent List | | |
| --- | --- | --- |
| Instrument: BD FACS Symphony A3 Lite | | |
| Antibody/Probe | **Fluorochrome** | **Vendor/Cat. No./Clone** |
| Mouse Anti-Human CD45 | BUV563 | BD/#748720/HI30 |
| Mouse IgG1, κ Isotype Control | BUV563 | BD/#612920/X40 |
| Mouse Anti-Human CD41a | BV786 | BD/#740975/HIP8 |
| Mouse IgG1, k Isotype Control | BV786 | BD/#563330/X40 |
| Mouse Anti-Human CD31 | PE | BD/#555446/WM59 |
| Mouse IgG1, κ Isotype Control | PE | BD/#555749/MOPC-21 |
