# Supplementary material for: Procoagulant effects of extracellular vesicles in patients with livedo reticularis
Source: Front Immunol. 2026 Sep 18;17:1940778. doi: 10.3389/fimmu.2026.1940778 (PMC13630680; doi:10.3389/fimmu.2026.1940778)
Supplement: Supplementary file 4 [file Table1.docx]

Supplementary table 1: Patient details.
